# Correlative Imaging of Three-Dimensional Cell Culture on Opaque Bioscaffolds for Tissue Engineering Applications

**DOI:** 10.1021/acsabm.3c00408

**Published:** 2023-09-01

**Authors:** Mone’t Sawyer, Josh Eixenberger, Olivia Nielson, Jacob Manzi, Cadré Francis, Raquel Montenegro-Brown, Harish Subbaraman, David Estrada

**Affiliations:** † Biomedical Engineering Doctoral Program, Boise State University, Boise, Idaho 83725, United States; ‡ Department of Physics, Boise State University, Boise, Idaho 83725, United States; § Center for Advanced Energy Studies, Boise State University, Boise, Idaho 83725, United States; ∥ Department of Chemical and Biological Engineering, University of Idaho, Moscow, Idaho 83844, United States; ⊥ School of Electrical Engineering and Computer Science, Oregon State University, Corvallis, Oregon 97331, United States; # Center for Atomically Thin Multifunctional Coatings, Boise State University, Boise, Idaho 83725, United States; ∇ Micron School for Materials Science and Engineering, Boise State University, Boise, Idaho 83725, United States; ○ Idaho National Laboratory, Idaho Falls, Idaho 83401, United States

**Keywords:** graphene foam, tissue engineering, correlative microscopy, microcomputed tomography, gold nanoparticles

## Abstract

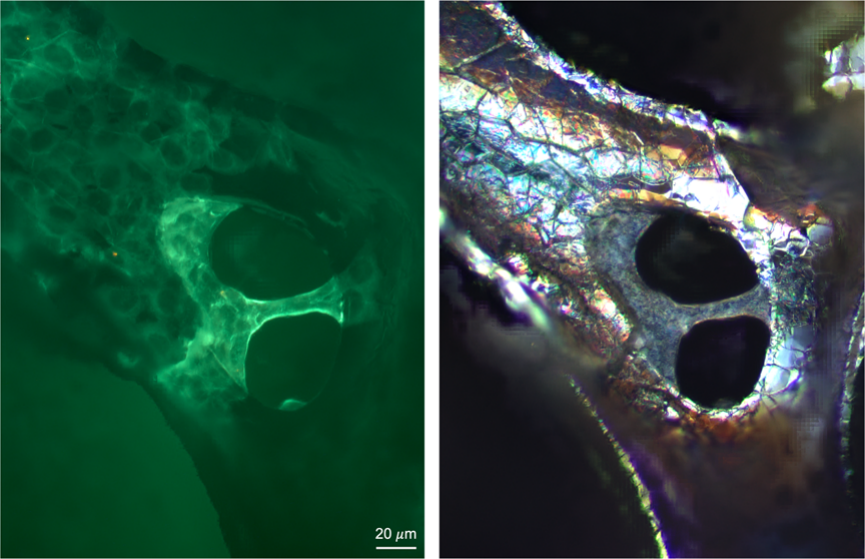

Three-dimensional (3D) tissue engineering (TE) is a prospective treatment that can be used to restore or replace damaged musculoskeletal tissues, such as articular cartilage. However, current challenges in TE include identifying materials that are biocompatible and have properties that closely match the mechanical properties and cellular microenvironment of the target tissue. Visualization and analysis of potential 3D porous scaffolds as well as the associated cell growth and proliferation characteristics present additional problems. This is particularly challenging for opaque scaffolds using standard optical imaging techniques. Here, we use graphene foam (GF) as a 3D porous biocompatible substrate, which is scalable, reproducible, and a suitable environment for ATDC5 cell growth and chondrogenic differentiation. ATDC5 cells are cultured, maintained, and stained with a combination of fluorophores and gold nanoparticles to enable correlative microscopic characterization techniques, which elucidate the effect of GF properties on cell behavior in a 3D environment. Most importantly, the staining protocol allows for direct imaging of cell growth and proliferation on opaque scaffolds using X-ray MicroCT, including imaging growth of cells within the hollow GF branches, which is not possible with standard fluorescence and electron microscopy techniques.

## Introduction

Articular cartilage damage is a frequent occurrence that can lead to osteoarthritis, the most prevalent joint disease and the leading cause of disability in the United States and other developed nations.^1,2^ Tissue engineering (TE), a prospective alternative treatment for this musculoskeletal disorder, aims to repair, maintain, or regenerate these damaged tissues; however, human tissues are complex in function, structural hierarchy, and scale, making it difficult to synthesize functional tissue in a lab that can be used for clinical treatments.^3−5^ Although there have been advances in tissue engineering, significant barriers remain regarding the ability to generate functional articular cartilage that imitates native cartilage both in structure and mechanical function.^6,7^

Over the past decade, articular cartilage tissue engineering has evolved from two-dimensional (2D) cell cultures grown on planar surfaces to culturing cells in complex three-dimensional (3D) architectures.^5,8^ The goal with next-generation bioscaffolds is to closely mimic the native environment of articular cartilage as it is known that the material properties of bioscaffolds can drive specific cell behaviors such as proliferation, differentiation, and extracellular matrix (ECM) production leading to tissue formation.^9−11^ In addition, bioscaffold properties can be used to deliver localized physical cues needed to stimulate tissue growth for engineering articular cartilage.^12^ Although biological materials such as collagen and alginate can simulate native extracellular matrix (ECM), they lack mechanical durability with a limited potential for improved functionality.^13,14^ Novel engineered biomaterials introduce a better mechanical integrity, mimicry of complex structures, and a high degree of control over material properties.^15^ New generation composite scaffolds such as poly(vinyl alcohol)-based hydrogels and polymeric/alginate composites exhibit more favorable mechanical properties; however, they do not address the limit of scalability and tunable control over cell behavior.^16,17^

Graphene and its derivatives have been established as superb scaffolds for cell culture with the dexterity to undergo long-term in vitro tissue engineering required for the growth of cartilage.^18−22^ Graphene foam (GF) is a porous 3D biocompatible substrate that is easily produced via chemical vapor deposition (CVD) on a nickel template.^22,23^ Its unique material properties, such as high electron mobility, excellent thermal conductivity, and high mechanical strength, can be harnessed to drive cell behavior, while synthesis via the CVD process lends to its scalability.^24−26^ Further, the microporous structure of GF facilitates nutrient exchange, and the high surface-to-volume ratio provides a favorable environment for long-term cell attachment and growth, making it an optimal candidate for next-generation 3D biomaterials.^27,28^

Although 3D environments are more conducive to functional tissue formation, characterization of cell proliferation and migration in these systems remains a challenge. Unlike 2D cell cultures, analyzing a single cell plane is not sufficient when working with 3D systems as it is important to assess the proliferation and migration of cells within the bioscaffold to determine the correlation between porosity, structure thickness, and pore interconnectivity.^29,30^ A high cell density and an even spatial distribution are associated with functional tissue formation; therefore, it is important to accurately evaluate cell attachment as well as cell distribution and density after seeding.^11^ Common characterization and analysis protocols such as transmission electron microscopy, scanning electron microscopy, and confocal fluorescence microscopy are tailored to analyzing cells in a 2D format, but these methods are not fully adapted to 3D analysis of the bioscaffolds internal structure and are limited by the bioscaffold’s opacity.^31^ Microcomputed tomography (MicroCT) techniques have been developed to study bioscaffold architecture without sample damage; however, direct imaging of the cells in a 3D environment is difficult due to their low contrast.^32,34^ This limits understanding of the mobility of cells in optically opaque bioscaffolds like GF, and to the best of our knowledge, a technique to assess cellular migration in the interior of GF bioscaffolds has not yet been realized. A previous study comparing the effect of different cell seeding methods within opaque poly(l-lactide-*co*-ε-caprolactone) based composite bioscaffolds utilized iron oxide nanoparticles to label cells in an attempt to investigate cellular infiltration; however, the technique proved difficult to quantify through MicroCT as the iron particles were a similar density as the ceramic components of the scaffold. Further, the efficacy of the labeling technique varied between cell donor lines, and the intracellular particle uptake was nonexistent 3 days after labeling.^32^

Here, we have demonstrated a method for labeling cells grown on GF bioscaffolds to quantify the effect of GF properties on the cellular spatial distribution in a 3D environment. We also highlight the limitations of planar analysis in 3D environments, emphasizing the importance of characterizing the cell behavior throughout the entire bioscaffold. The novelty of this research lies in the development of labeling and correlative imaging techniques using a conjugated fluorophore to study cellular spatial distribution on GF bioscaffolds in conjunction with MicroCT techniques. The proposed labeling technique can be applied to other bioscaffold materials, offering a promising method for quantifying cell migration in various opaque 3D architectures.

## Results

### Graphene Foam Growth and Characterization

**Figure 1:**
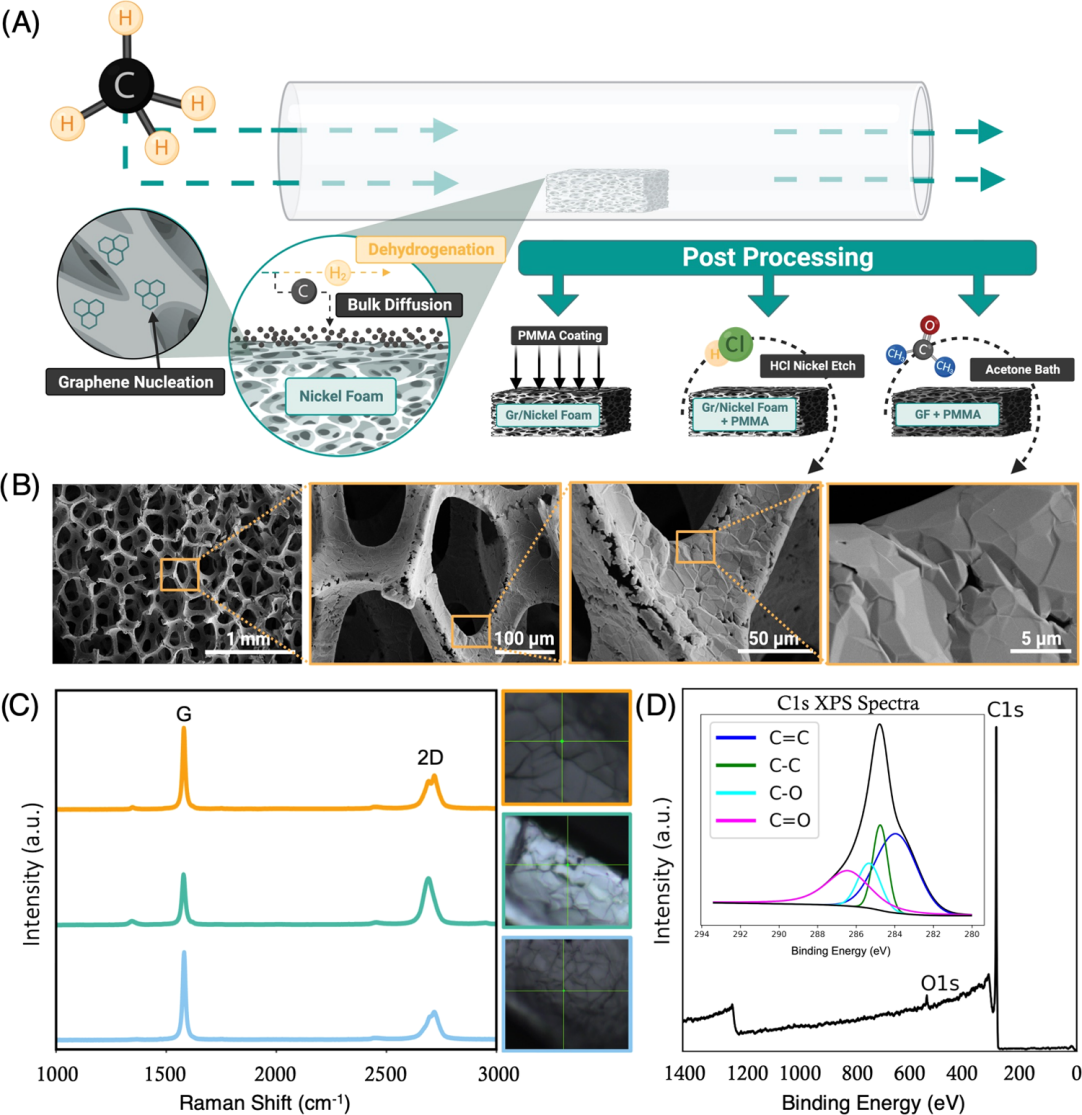
GF syntheses and characterization. (A) GF is synthesized via CVD on a nickel foam template. Gr/Nickel foam is coated in PMMA to maintain integrity during a 3 M HCl bath to dissolve the nickel template. After nickel dissociation, PMMA is dissolved in an acetone bath. (B) SEM micrographs show the bulk structure and wrinkled topography of CVD GF with increasing magnification. (C) Raman spectroscopy indicates graphitic quality, with the characteristic G and 2D peaks seen in graphitic materials. (D) X-ray spectra confirm the complete dissociation of the nickel foam template.

An open-source CVD furnace was utilized to synthesize GF bioscaffolds using a nickel foam template (Figure 1A).^23^ Following synthesis, the graphene/nickel foam substrates are etched in 3 M hydrochloric acid until complete dissociation of the nickel. After etching, scanning electron microscopy (SEM) was used to characterize the superficial microstructure of GF (FEI Teneo Field Emission Scanning Electron Microscope). Micrographs in Figure 1B show the macroporous structure and wrinkled topography of GF with increasing magnification as well as microcracks in the branches due to the dissociation and internal removal of the nickel foam template. GF was further analyzed with Raman spectroscopy to quantify the graphitic nature of our CVD GF (Figure 1C). Raman spectra were compared at three separate locations across a single sample, further confirming that the quality of GF is consistent throughout the bulk. Each of the separate Raman spectra exhibits the characteristic G (∼1585 cm^–1^) and 2D (∼2700 cm^–1^) peaks typical of graphitic materials, whereas the absence or low intensity of the characteristic D (∼1350 cm^–1^) peak indicates the low defect density of the GF.^26^ Additional analysis was done using X-ray photoelectron spectroscopy (XPS), which verified complete dissociation of the nickel foam template (Figure 1D).

### Visualization of Cells on GF Using Fluorescence and Reflected Light Imaging

**Figure 2:**
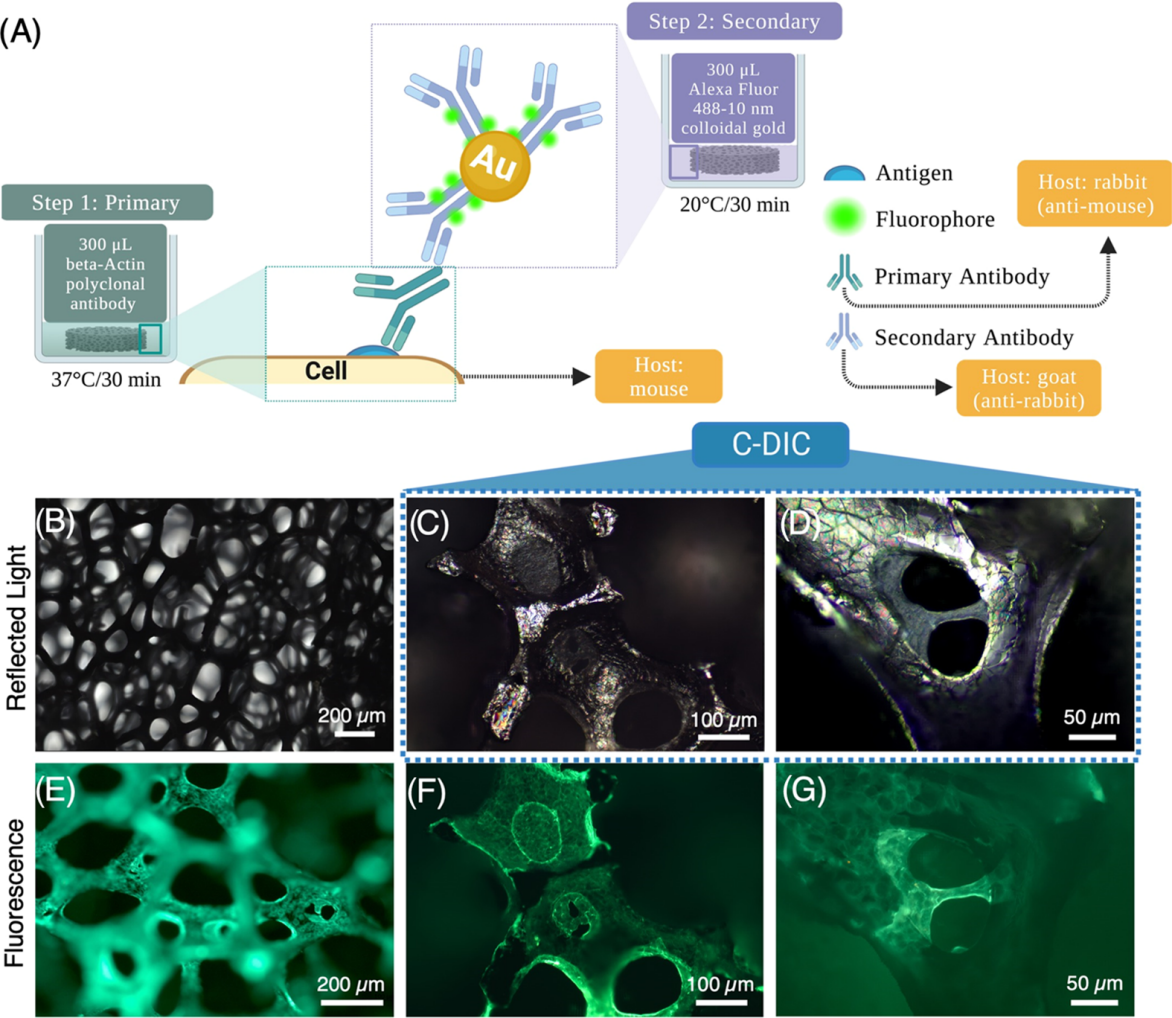
Indirect labeling of ATDC5 cells on GF bioscaffolds. (A) Labeling procedure and incubation parameters for double labeling with β-Actin polyclonal antibody and Goat anti-Rabbit IgG (H + L) Secondary Antibody Alexa Fluor 488–10 nm colloidal gold. (B) Reflected light micrographs exhibit GF’s opaque quality with limited z-resolution. (C–D) C-DIC shows the wrinkled structure of GF and ATDC5 cells spanning pores colocalized with fluorescence images (F,G). (E–G) Fluorescence micrographs with fluorescently labeled actin allow for characterization of ATDC5 cell attachment on the GF surface.

A schematic diagram showing the process for the indirect labeling of ATDC5 cells is shown in Figure 2A. After 7 days of cell growth, fixation and permeabilization of the cells were performed. Samples were first stained with a primary polyclonal antibody (BS-0061R) targeting β actin, followed by staining with a secondary antibody that is conjugated to 10 nm colloidal gold and Alexa Fluor 488 (AB_2536179). In Figure 2C,D, samples were imaged using circular polarized light-differential interference contrast (C-DIC), a reflected light technique, which converts gradients in the specimen optical path into sample amplitude differences and allows for the visualization of the wrinkled topography of GF bioscaffolds.^36^ Fluorescently labeled actin allowed for the quantification of anchorage-dependent ATDC5 cell behavior on the superficial GF surface, where cells spanning GF pores can be visualized with both reflected light and fluorescence imaging.

**Figure 3:**
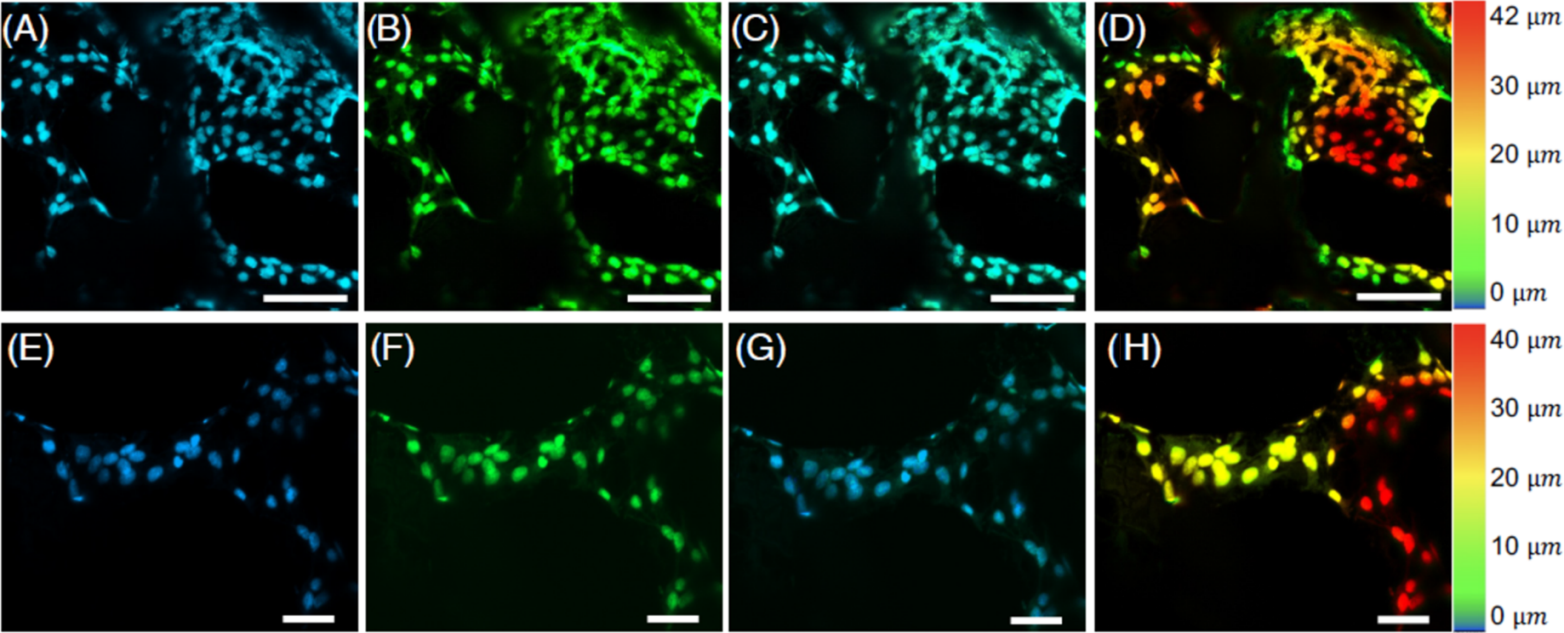
Confocal fluorescence imaging of ATDC5 cells on GF bioscaffolds following 7 days of cell growth, illustrating how the thickness of the GF is a constraint to full sample analysis. (A–D) Immunofluorescent micrographs depicting a 10× maximum intensity projection of a 42 μm Z-stack: (A) Nucleus labeled with NucBlue Live ReadyProbes(scale bar = 100 μm); (B) double-labeled phalloidin with β-Actin polyclonal antibody and Goat anti-Rabbit IgG (H + L) Secondary Antibody Alexa FluorTM 488–10 nm colloidal gold; (C) merged NucBlue and actin; (D) color-coded heatmap projection of cellular z-position on GF branches. (E, F) Immunofluorescent micrographs depicting a 10× maximum intensity projection of a 40 μm Z-stack of a smaller region of interest within the volume of (A–D) (scale bar = 50 μm).

Cell attachment was further analyzed through confocal immunofluorescence imaging, as demonstrated in Figure 3. The reconstruction of z-stacks using the maximum intensity projections (MIP) fusion method makes clear the constraints that arise from GF thickness when imaging using this standard optical technique. In Figure 3D,H, heatmaps with color-coded representations illustrate the attainable thickness range using a 10× objective while extraneous out-of-focus illumination from adjacent planes in the sample.

### Evaluation of GF and Cell-GF Interactions Using Scanning Electron Microscopy

**Figure 4:**
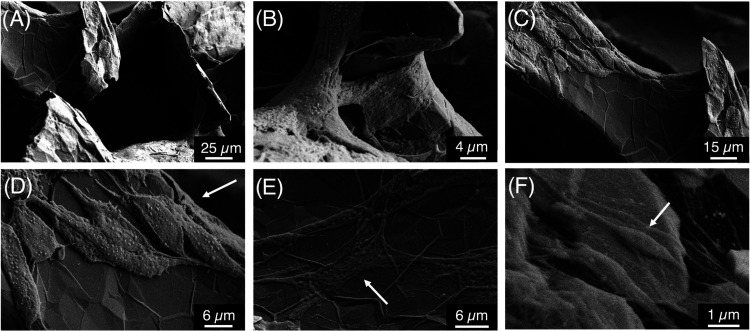
Scanning electron micrographs of adherent ATDC5 cells on GF bioscaffolds. (A–F) SEMs confirm that the gold nanoparticles were conjugated with the secondary antibody, allowing for the visualization of cell attachment and morphology in addition to the surface structure of GF. Arrows indicate bipolar and multipolar fibroblastic morphologies of cells across the scaffold.

Scanning electron micrographs allowed for the quantification of cell attachment and morphology on the surface of GF without any further processing due to the gold nanoparticle–antibody conjugates used to label actin. Traditionally, cells are sputter-coated with a layer of conductive material for this type of analysis.^28,37^ Although the sputtering technique allows for the visualization of cells spanning GF pores and some surface interactions, it is limited in its ability to resolve singular cellular interactions with the rough GF surface. The indirect labeling technique with colloidal gold resulted in high-resolution micrographs where several cell–graphene interactions can be visualized, as seen in Figure 2. Cells wrap around GF branch structures (Figure 4A,B), exhibiting both bipolar (Figure 4D,F) and multipolar (Figure 4E) morphologies indicative of fibroblastic cells.^38^

### Microcomputed Tomography Characterization of Cellular Distribution within GF Bioscaffolds

**Figure 5:**
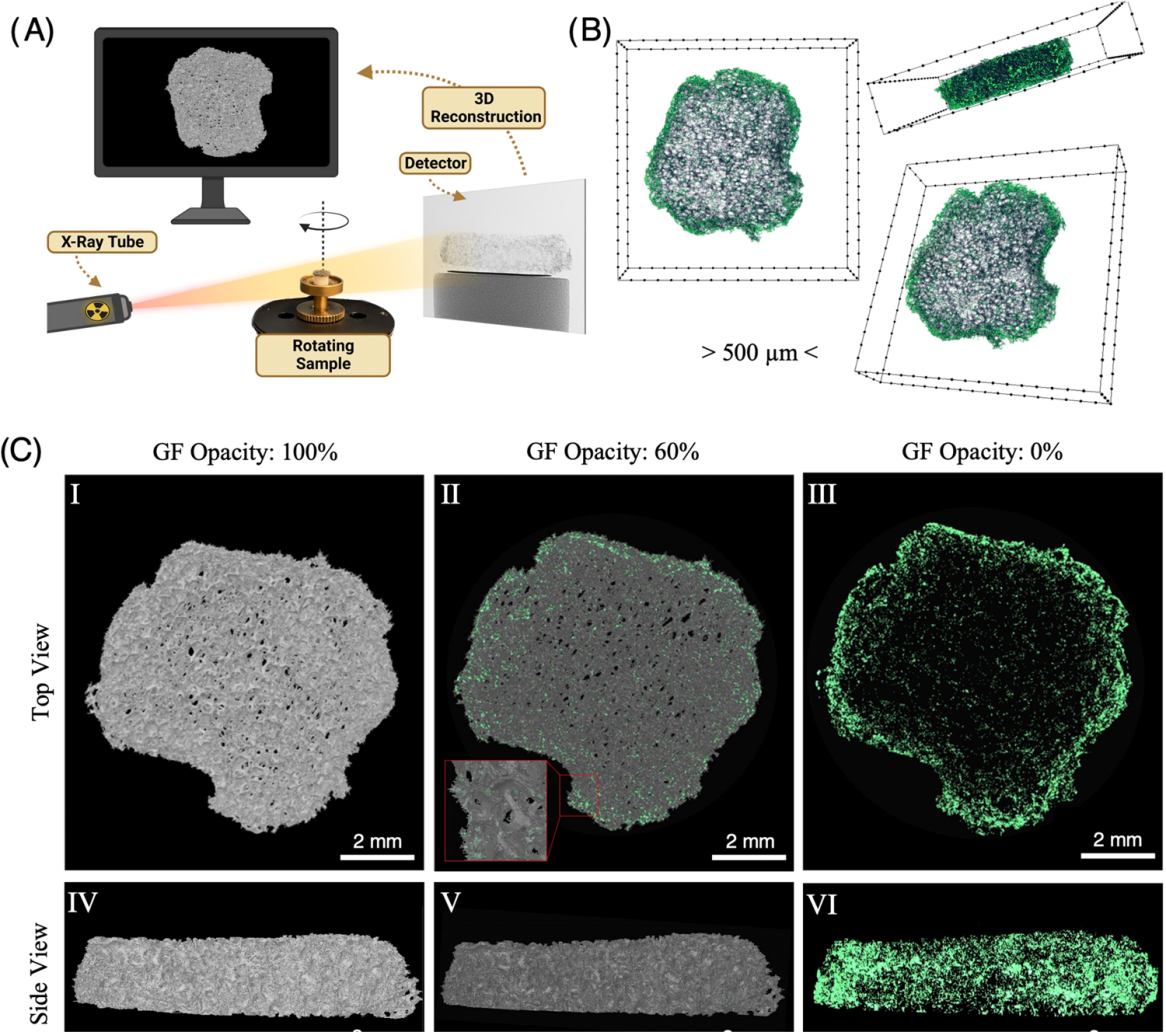
MicroCT analysis of indirectly labeled ATDC5 cells on GF. (A) Schematic illustration showing the basis of MicroCT. Cone beam X-rays travel from the source to the detector through the sample where a grayscale projection image is acquired at each rotation. (B) CTVox 3D projection of a reconstructed 2D slice from MicroCT acquisition where cells (green) have a higher density at the scaffold edge. (C) 3D reconstruction of GF (gray) and cells (green). A decrease in GF opacity (II, III, V, and VI) allows for visualization of cellular spatial distribution in three dimensions.

MicroCT (Bruker, Skyscan 1172, Belgium) was performed on bare GF as well as GF with ATDC5 cells labeled with the antibody–gold nanoparticle conjugates to determine the internal structure of GF and the spatial distribution of the cells within the 3D environment (Figure 5A). NRecon software was used to reconstruct the angular projections into cross-sectional slices for 3D reconstruction and volumetric analysis using the same attenuation range for all samples. The reconstructed images were then processed using Skyscan’s CT Analyzer (CTan) to binarize the 2D images for 3D reconstruction and volumetric analysis (Figure 5A). The binarized 3D model of bare GF was calculated to have an average structure thickness (St.Th) of 8.625 ± 2.54 μm, a surface area to volume ratio of 368.55 mm^–1^, and an object volume to total volume (Obj. V/TV) ratio 2.561% corresponding to a porosity of 97.439%. Several studies have shown that cellular behavior is influenced by fluid flow and nutrient diffusion in 3D culture environments, and the high porosity of our GF is advantageous such that it facilitates the exchange of waste products for fresh nutrients during longer culture periods.^35^

**Figure 6:**
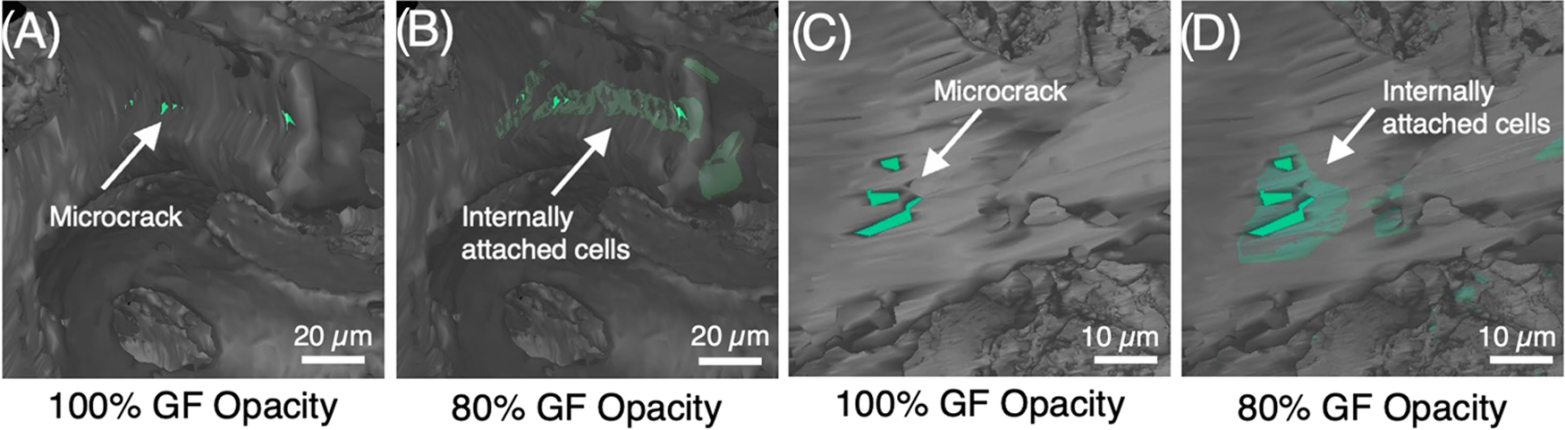
MicroCT analysis of indirectly labeled ATDC5 cells within GF branches. Locating a microcrack in the branch sidewall and reducing the opacity of the GF from 100 to 80% (A–D) allows for visualization of internal branch cell attachment and migration through microcracks.

Visual analysis was done using CTVox software, which takes the reconstructed 2D images from the scans and projects them in three dimensions, and CTVol software, which takes the binarized 2D cross-sectional slices and renders a complete 3D reconstruction. In both the projection and 3D reconstruction, the conjugated gold nanoparticles enabled the optical segmentation and false coloring of the cells (green) and the scaffold (gray). In Figure 5B, the CTVox projections enable visualization of how the cells are distributed throughout the bulk of the GF and reveal the cell density is higher at the edges of the scaffold. The 3D reconstruction in Figure 5C allows for the quantification of the spatial distribution of cells within the internal structure of the GF. Cells can be visualized more clearly by decreasing GF opacity, and although a side view with the GF set to 0% opacity indicates that cells are evenly dispersed throughout the bulk of the scaffold, a top view indicates that cell density is higher toward the outer edge, which is indicative of GF’s hydrophobicity, and a characteristic in agreement with suspected observations of fluorescence micrographs and confirmed CTVox projections. In addition, our CVD synthesis method for GF bioscaffolds results in microcracks in the branch sidewall as seen in SEM (Figure 1B), leaving the internal branch structure open for cell migration, attachment, and proliferation but difficult to confirm. Traditional methods of sputtering for electron microscopy would not allow for the characterization of that behavior, whereas antibody staining with colloidal gold enabled visualization of cell migration within the branch structure using MicroCT techniques (Figure 6). Discovery of this behavior indicates that, with CVD GF bioscaffolds, the surface area is not necessarily sacrificed for increased porosity.

## Discussion

Indirect labeling with the conjugated fluorophore allowed us to utilize fluorescence microscopy, and the conjugated gold enabled characterization of cell attachment and morphology on the superficial plane of our GF bioscaffolds using SEM. However, it is apparent that planar analysis alone is not sufficient in 3D environments. Common 3D scaffolds typically rely on seeding via the “drop-on” method, with most cells remaining on the superficial seeded surface of the scaffold, resulting in poor cellular penetration and spatial distribution.^39,40^ This study shows that the external surface alone does not accurately represent cell activity in the bulk. We have demonstrated a simple labeling technique that can be utilized for each of the aforementioned characterization modes as well as MicroCT without any additional processing to study this behavior and its correlation to the volumetric and material properties of the GF. MicroCT scans and 3D rendering of porous structures are computationally expensive and time-consuming. However, we have demonstrated this staining protocol can be verified prior to MicroCT scans by using fluorescence microscopy or SEM. Additionally, the labeling can be verified visually in the MicroCT before scanning by using the real-time display utilizing the same X-ray settings for scanning bare GF (Figure S1A).

Through this work, we have found that cellular distribution within GF bioscaffolds is not limited to the superficial seeding surface, despite its hydrophobicity, and that cellular distribution, while limited, takes place throughout the bulk of the scaffold without any external induction such as dynamic flow or rotational seeding. In addition, this is the first instance in which cellular attachment within the internal structure of the graphene branch has been visualized. Confirmation of this activity indicates that GF bioscaffolds can be further engineered to utilize this feature to better suit certain cell types and tissue organization. Through the CVD process, the GF can be tailored to achieve the desired porosity and structure thickness of the bioscaffolds by varying the geometric parameters of the nickel foam template. Furthermore, since cells can migrate to the internal branch structures of the GF, this finding demonstrates that cells may attach to either side of the GF branches, which increases the effective surface area of the bioscaffolds creating a more robust tissue coverage. By determining how the structure of GF bioscaffolds affects cell behavior, bioscaffolds can be designed to facilitate cell organization that corresponds to articular cartilage tissue engineering.

The ability to quantify cell migration in opaque bioscaffolds is a major gap in 3D tissue engineering, as scaffolds with increased mechanical strength generally have increased opaqueness. There is no one way to ensure a certain seeding procedure is optimal across different scaffold materials as it is widely unique to the scaffold properties and architecture; however, the imaging protocol utilized is not limited to GF and could be adapted to any bioscaffold with an optical density that can be segmented from gold.

## Conclusions

GF offers unique material properties that can be taken advantage of to influence cell behavior, making GF an ideal candidate for next-generation 3D biomaterials. However, characterizing cell proliferation and migration within such 3D systems remains a challenge. To address this gap, we developed a labeling technique using a conjugated fluorophore to study cellular spatial distribution on GF bioscaffolds using MicroCT techniques. This approach allowed us to visualize cells within the internal branch structures of GF, providing insights into cell migration, attachment, and proliferation within the 3D environment, the feasibility of which would be unattainable through conventional optical methods of characterization.

The results demonstrate that cells exhibit an even spatial distribution throughout the bulk of the GF bioscaffold, despite using the “drop-on” method of cellular seeding. This finding opens up new possibilities for engineering GF bioscaffolds to utilize this feature for specific cell types and tissue organization. The CVD synthesis method for GF offers the potential to tailor GF physical properties in order to achieve high porosity and structure thickness for tissue engineering applications.

Additionally, this study highlights the limitations of relying solely on planar analysis in 3D environments and emphasizes the importance of characterizing cell behavior throughout the entire bioscaffold. The presented labeling technique can be applied to other bioscaffold materials, offering a promising method for quantifying cell migration in various opaque 3D architectures.

Overall, our results offer a new tool to probe the fundamental role of the GF’s 3D structure on cell behavior. The combination of GF’s unique material properties and the proposed labeling technique holds great potential for future advancements in tissue engineering and regenerative medicine applications. The findings presented in this paper pave the way for further studies in the field, aiming to refine tissue engineering strategies and ultimately improve the treatment options available for patients suffering from articular cartilage damage and osteoarthritis.

## Materials and Methods

### CVD Graphene Foam

An open-source CVD furnace was used to synthesize GF bioscaffolds using a 1.2 mm thick nickel (Ni) foam template.^23^ The Ni foam cut was cut (3 cm × 8 cm) and placed inside a 2 in. quartz tube. The Ni foam was annealed for 30 min at 1000 °C and graphene was grown under CH_4_ flow at 1000 °C for 50 min before undergoing a cooling cycle to room temperature. The Ni/graphene foam composite was coated with poly(methyl methacrylate) (PMMA) and dried for 24 h to maintain the structural integrity of the foam during postprocessing. Coated Ni/graphene foam substrates were etched in 3 M HCl on a 60 °C hot plate until the nickel foam template was completely dissociated, at which point the PMMA was dissolved with acetone. The resulting GF was rinsed with Millipore water, dried, and cut into circles with an 8 mm diameter before characterization.

### Bare Graphene Foam Characterization

The superficial microstructure and surface topography of the GF bioscaffolds were evaluated via scanning electron microscopy (FEI Teneo Field Emission Scanning Electron Microscope). SEM samples were attached to the SEM post with double-sided carbon tape, and electron micrographs were collected at 5.00 kV and 25 pA utilizing an Everhart–Thornley detector (ETD). Micrographs from SEM were used to manually measure the average pore size with ImageJ software for comparison with MicroCT volumetric analysis (Figure S2). Raman spectroscopy (Horiba Scientific LabRAM HR Evolution Raman Microscope) and X-ray photoelectron spectroscopy (XPS) were performed to determine the graphitic nature of the GF and confirm complete dissociation of the nickel foam template. X-ray Photoelectron Spectroscopy (XPS) measurements were performed with a Physical Electronics ESCA 5600 in the Atomic Films Laboratory at Boise State University using an Al kα excitation source. Low-resolution survey scans of the surface were performed initially to measure relative atomic concentrations. The sample was found to contain 91.04% Carbon (C 1s) and 8.96% Oxygen (O 1s). Data were analyzed with MultiPak 9.6 software, and all spectra were referenced to the C 1s peak (284.8 eV) for adventitious carbon. The survey region ranged from 0 to 1400 eV with a step size of 0.400 eV. Elemental peaks in the survey spectra were initially identified using the software’s automatic peak identification feature and verified using the Handbook of X-ray Photoelectron Spectroscopy.^41^ Peak fitting on the high-resolution spectra utilized a Gaussian–Lorentzian fit with a Smart background. A high-resolution scan of the C 1s peak was obtained for chemical analysis using a pass energy of 23.50 eV and a step size of 025 eV. As observed in the inset of Figure 1d, the C 1s spectra include four peaks with binding energies of 283.94, 284.75, 285.32, and 286.44 eV associated with C=C (sp^2^ hybridization), C–C (sp^3^ hybridization), C–O, and C=O respectively.

### Microcomputed Tomography of GF

Bulk structural characterization was evaluated via microcomputed tomography (SkyScan 1172 X-ray MicroCT). Briefly, GF samples were mounted onto a porous polyethylene pipet filter with double-sided tape, where a drop of 70% ethanol was placed atop the GF to ensure mounting to the tape without needing to add pressure to the top of the sample. After drying, the GF/filter was placed upright on the sample holder and secured into place with double-sided tape to eliminate scan artifacts due to random movement.^35^ Scan acquisition on bare and labeled GF bioscaffolds was conducted with a 26 kV source voltage, 145 μA current, and 2650 ms exposure time. Scan parameters were defined with a step size of 0.25°, ten-frame averaging, and 2.24 μm pixel size. NRecon software was used to reconstruct the angular projections into cross-sectional slices for 3D reconstruction and volumetric analysis with an attenuation value for all samples from 0 to 0.5000. Bruker Skyscan CT Analyzer (CTan) software was used to binarize the 2D images; bare GF scans were binarized with a threshold value of 18–115 range on the contrast scale, while labeled samples were segmented into two contrast scales: (1) the gold nanoparticle-labeled cells (115–255) and (2) for the GF (18–115), for 3D reconstruction and volumetric analysis. GF structure thickness, surface area to volume ratio, object to total volume ratio, and the corresponding porosity were calculated using CTan software. 3D reconstruction of the GF environment was qualitatively analyzed using CTVol software, and shadow projections were visualized using CTVox software. To highlight the cells versus the GF, cells were false-colored to green with the transfer function editor using the linear interpolation method in CTVox^42^ (Figure S1B) and by overlaying the falsely colored 3D models in CTVol.

### Preparing GF for Cell Culture

The GF bioscaffolds used for cell culture were cut from the same sheet of Ni foam and synthesized in the same batch to ensure consistency across the substrates. To prepare our GF bioscaffolds for ATDC5 (Sigma-Aldrich, St. Louis, MO) cell culture, they were sterilized with 70% ethanol prior, rinsed with DPBS to conditioning them in growth media (F12/Dulbecco’s Modified Eagle Medium (DMEM/F12), 5% (v/v) fetal bovine serum (FBS), and 1% (v/v) penicillin/streptomycin) for 24 h before seeding them with cells. Additionally, an anti-adherence rinsing solution (STEMCELL technologies) was used in the well plates to prevent cell growth on the cultureware containing the GF and to promote cell growth on the scaffold.

### Cell Culture

Conditioned GF bioscaffolds were seeded with ATDC5 chondrocyte progenitor cells by pipetting 500 μL of cell suspension (5 × 10^6^ cells) to the topside of the GF. They were then incubated for 7 days in growth media (GM) at 37 °C and 5% CO_2_. Cell growth was monitored with transmitted light microscopy and GM changed daily.

Cells were fixed on GF bioscaffolds with 0.2% paraformaldehyde, permeabilized with 0.1% Triton-X and directly labeled with β-Actin polyclonal antibody (Thermo Fisher Scientific) at a concentration of 1 μg/mL before incubation at 37 °C for 30 min. Bioscaffolds were rinsed 10 times with PBS diluted in nanopure water (10:1), then stained with a 30 μg/mL concentration of Goat anti-Rabbit IgG (H + L) Secondary Antibody Alexa Fluor 488–10 nm colloidal gold, incubated at 20 °C in the dark for 30 min, and then rinsed 10 times in diluted PBS and dried. The diluted PBS rinsing steps ensure that salt crystals that form from drying PBS do not affect SEM or MicroCT acquisition (Figure S3). Samples were imaged with a Zeiss Axio Imager, M2 upright microscope fitted with a Zeiss Colbri 5 LED light source, a fluorescence filter cube, a C-DIC slider, and an Axiocam 305 color digital camera (Carl Zeiss, Inc.). Z-stack images using reflected light, fluorescence, and C-DIC were acquired from the EC Epiplan 50×/0.7, 20×/0.4, 10×/0.25 HD M27 objectives. Samples were then imaged with a Zeiss LSM 900 confocal system combined with a Zeiss Axio Observer.Z1. Confocal Z-stack micrographs were acquired using the Plan-Apochromat 10*x*/0.45 objective with laser wavelengths of 405 and 488 nm at a laser power of 1.2 and 1%, respectively. Image processing was performed with ZEN imaging software, with the exception of the maximum intensity projections for the Z-stacks, which were acquired with FIJI software. GF–cell interactions with the GF surface were analyzed with SEM and MicroCT with the same mounting and scanning methods used for the bare GF samples.

## Supporting Information Available

Methods for MicroCT imaging and transfer function editing. (A) X-ray comparison between bare GF and GF with labeled cells was used to confirm labeling protocol before acquisition where labeled cells are darker in color due to an increase in density from the colloidal gold. (B) Transfer function editing with attenuation histogram for CTVox (Opacity, RGB) shadow projections where cells are false-colored to gold and GF is colored to gray/black; pore size and structure thickness measurements of bare GF. (A) Scanning electron micrograph of bare GF indicating the direction that pores were measured utilizing ImageJ discerning between volumetric (blue) versus surface pores (red). (B) Structure thickness measurement of GF sidewall from SEM. (C) Histograms of average pore size for volumetric pores, surface pores, and all pores; when not rinsed with diluted DPBS, salt crystals cover GF branches following cell culture. Scale Bars: (A) 50 μm, (B) 50 μm, (C) 25 μm, and (D) 5 μm (PDF)
MicroCT video of GF and cells with the opacity of the GF gradually decreasing shows that cellular density is significantly increased at the scaffold edge. As the outer edges of the GF are typically subject to handling during experiments, a cross-section cut through the center shows the bulk internal porous structure that is difficult to visualize at the outer edge alone. Any pixels not fully attached to GF structure are caused by the tape and filter used for mounting (MOV)
MicroCT video of ATDC5 cell distribution in 3D space allows for full quantification of how cells are spatially arranged when the scaffold is set at 0% opacity, confirming that cells migrate through the bulk of the scaffold despite using the “drop-on” method of seeding on the superficial surface (MOV)

## Supplementary Material

mt3c00408_si_001.pdf

mt3c00408_si_002.mov

mt3c00408_si_003.mov

## References

[ref1] Wallace I. J.; Worthington S.; Felson D. T.; Jurmain R. D.; Wren K. T.; Maijanen H.; Woods R. J.; Lieberman D. E. Knee Osteoarthritis Has Doubled in Prevalence since the Mid-20th Century. Proc. Natl. Acad. Sci. U.S.A. 2017, 114 (35), 9332–9336. 10.1073/pnas.1703856114. 28808025 PMC5584421

[ref2] Armiento A. R.; Alini M.; Stoddart M. J. Articular Fibrocartilage - Why Does Hyaline Cartilage Fail to Repair?. Adv. Drug Delivery Rev. 2019, 146, 289–305. 10.1016/j.addr.2018.12.015. 30605736

[ref3] Khademhosseini A.; Langer R. A Decade of Progress in Tissue Engineering. Nat. Protoc. 2016, 11 (10), 1775–1781. 10.1038/nprot.2016.123. 27583639

[ref4] O’Brien F. J. Biomaterials & Scaffolds for Tissue Engineering. Mater. Today 2011, 14 (3), 88–95. 10.1016/S1369-7021(11)70058-X.

[ref5] Zhang L.; Hu J.; Athanasiou K. A. The Role of Tissue Engineering in Articular Cartilage Repair and Regeneration. Crit. Rev. Biomed. Eng. 2009, 37, 1–57. 10.1615/critrevbiomedeng.v37.i1-2.10. 20201770 PMC3146065

[ref6] Francis S. L.; Di Bella C.; Wallace G. G.; Choong P. F. M. Cartilage Tissue Engineering Using Stem Cells and Bioprinting Technology—Barriers to Clinical Translation. Front. Surg. 2018, 5, 70 10.3389/fsurg.2018.00070. 30547034 PMC6278684

[ref7] Armstrong J. P. K.; Pchelintseva E.; Treumuth S.; Campanella C.; Meinert C.; Klein T. J.; Hutmacher D. W.; Drinkwater B. W.; Stevens M. M. Tissue Engineering Cartilage with Deep Zone Cytoarchitecture by High-Resolution Acoustic Cell Patterning. Adv. Healthcare Mater. 2022, 11, 2200481 10.1002/adhm.202200481. PMC7614068 35815530

[ref8] Kwon H.; Brown W. E.; Lee C. A.; Wang D.; Paschos N.; Hu J. C.; Athanasiou K. A. Surgical and Tissue Engineering Strategies for Articular Cartilage and Meniscus Repair. Nat. Rev. Rheumatol. 2019, 15, 550–570. 10.1038/s41584-019-0255-1. 31296933 PMC7192556

[ref9] Stampoultzis T.; Karami P.; Pioletti D. P. Thoughts on Cartilage Tissue Engineering: A 21st Century Perspective. Curr. Res. Transl. Med. 2021, 69 (3), 103299 10.1016/j.retram.2021.103299. 34192658

[ref10] Breuls R. G. M.; Jiya T. U.; Smit T. H. Scaffold Stiffness Influences Cell Behavior: Opportunities for Skeletal Tissue Engineering. Open Orthop. J. 2008, 2, 103–109. 10.2174/1874325000802010103. 19478934 PMC2687114

[ref11] Cámara-Torres M.; Sinha R.; Scopece P.; Neubert T.; Lachmann K.; Patelli A.; Mota C.; Moroni L. Tuning Cell Behavior on 3d Scaffolds Fabricated by Atmospheric Plasma-Assisted Additive Manufacturing. ACS Appl. Mater. Interfaces 2021, 13 (3), 3631–3644. 10.1021/acsami.0c19687. 33448783 PMC7880529

[ref12] Litowczenko J.; Woźniak-Budych M. J.; Staszak K.; Wieszczycka K.; Jurga S.; Tylkowski B. Milestones and Current Achievements in Development of Multifunctional Bioscaffolds for Medical Application. Bioact. Mater. 2021, 2412–2438. 10.1016/j.bioactmat.2021.01.007. 33553825 PMC7847813

[ref13] Beck E. C.; Barragan M.; Tadros M. H.; Gehrke S. H.; Detamore M. S. Approaching the Compressive Modulus of Articular Cartilage with a Decellularized Cartilage-Based Hydrogel. Acta Biomater. 2016, 38, 94–105. 10.1016/j.actbio.2016.04.019. 27090590 PMC4903909

[ref14] Sun J.; Tan H. Alginate-Based Biomaterials for Regenerative Medicine Applications. Materials 2013, 6, 1285–1309. 10.3390/ma6041285. 28809210 PMC5452316

[ref15] Bajaj P.; Schweller R. M.; Khademhosseini A.; West J. L.; Bashir R. 3D Biofabrication Strategies for Tissue Engineering and Regenerative Medicine. Annu. Rev. Biomed. Eng. 2014, 16, 247–276. 10.1146/annurev-bioeng-071813-105155. 24905875 PMC4131759

[ref16] Barbon S.; Contran M.; Stocco E.; Todros S.; Macchi V.; De Caro R.; Porzionato A. Enhanced Biomechanical Properties of Polyvinyl Alcohol-Based Hybrid Scaffolds for Cartilage Tissue Engineering. Processes 2021, 9, 730 10.3390/pr9050730.

[ref17] Moutos F. T.; Guilak F. Composite Scaffolds for Cartilage Tissue Engineering. Biorheology 2008, 45, 501–512. 10.3233/BIR-2008-0491. 18836249 PMC2727640

[ref18] O’Brien F. J. Biomaterials & Scaffolds for Tissue Engineering. Mater. Today 2011, 14, 88–95. 10.1016/S1369-7021(11)70058-X.

[ref19] Tasnim N.; Thakur V.; Chattopadhyay M.; Joddar B. The Efficacy of Graphene Foams for Culturing Mesenchymal Stem Cells and Their Differentiation into Dopaminergic Neurons. Stem Cells Int. 2018, 2018, 1–12. 10.1155/2018/3410168. PMC6008666 29971110

[ref20] Kenry; Lee W. C.; Loh K. P.; Lim C. T. When Stem Cells Meet Graphene: Opportunities and Challenges in Regenerative Medicine. Biomaterials 2018, 236–250. 10.1016/j.biomaterials.2017.10.004. 29195230

[ref21] Lee S. K.; Kim H.; Shim B. S. Graphene: An Emerging Material for Biological Tissue Engineering. Carbon Lett. 2013, 14 (2), 63–75. 10.5714/CL.2013.14.2.063.

[ref22] Amani H.; Mostafavi E.; Arzaghi H.; Davaran S.; Akbarzadeh A.; Akhavan O.; Pazoki-Toroudi H.; Webster T. J. Three-Dimensional Graphene Foams: Synthesis, Properties, Biocompatibility, Biodegradability, and Applications in Tissue Engineering. ACS Biomater. Sci. Eng. 2019, 5, 193–214. 10.1021/acsbiomaterials.8b00658. 33405863

[ref23] Williams-Godwin L.; Brown D.; Livingston R.; Webb T.; Karriem L.; Graugnard E.; Estrada D. Open-Source Automated Chemical Vapor Deposition System for the Production of Two- Dimensional Nanomaterials. PLoS One 2019, 14 (1), e0210817 10.1371/journal.pone.0210817. 30650151 PMC6334948

[ref24] Balandin A. A.; Ghosh S.; Nika D. L.; Pokatilov E. P. Thermal Conduction in Suspended Graphene Layers. Fullerenes, Nanotubes Carbon Nanostruct. 2010, 18, 474–486. 10.1080/1536383X.2010.487785.

[ref25] Novoselov K. S.; Geim A. K.; Morozov S. V.; Jiang D.; Katsnelson M. I.; Grigorieva I. V.; Dubonos S. V.; Firsov A. A. Two-Dimensional Gas of Massless Dirac Fermions in Graphene. Nature 2005, 438 (7065), 197–200. 10.1038/nature04233. 16281030

[ref26] Saeed M.; Alshammari Y.; Majeed S. A.; Al-Nasrallah E. Chemical Vapour Deposition of Graphene—Synthesis, Characterisation, and Applications: A Review. Molecules 2020, 25, 3856 10.3390/molecules25173856. 32854226 PMC7503287

[ref27] Yocham K. M.; Scott C.; Fujimoto K.; Brown R.; Tanasse E.; Oxford J. T.; Lujan T. J.; Estrada D. Mechanical Properties of Graphene Foam and Graphene Foam—Tissue Composites. Adv. Eng. Mater. 2018, 20 (9), 1800166 10.1002/adem.201800166. 30581324 PMC6301055

[ref28] Frahs S. M.; Reeck J. C.; Yocham K. M.; Frederiksen A.; Fujimoto K.; Scott C. M.; Beard R. S.; Brown R. J.; Lujan T. J.; Solov’yov I. A.; Estrada D.; Oxford J. T. Prechondrogenic ATDC5 Cell Attachment and Differentiation on Graphene Foam; Modulation by Surface Functionalization with Fibronectin. ACS Appl. Mater. Interfaces 2019, 11 (45), 41906–41924. 10.1021/acsami.9b14670. 31639302 PMC6858527

[ref29] Cengiz I. F.; Oliveira J. M.; Reis R. L. Micro-CT - A Digital 3D Microstructural Voyage into Scaffolds: A Systematic Review of the Reported Methods and Results. Biomater. Res. 2018, 22, 28 10.1186/s40824-018-0136-8. 30275969 PMC6158835

[ref30] Temple J.; Velliou E.; Shehata M.; Lévy R.; Gupta P. Current Strategies with Implementation of Three-Dimensional Cell Culture: The Challenge of Quantification. Interface Focus 2022, 12 (5), 20220019 10.1098/rsfs.2022.0019. 35992772 PMC9372643

[ref31] Hollister S. J. Porous Scaffold Design for Tissue Engineering (Vol 4, Pg 518, 2005). Nat. Mater. 2006, 5 (7), 590 10.1038/nmat1683. 16003400

[ref32] Palmroth A.; Pitkänen S.; Hannula M.; Paakinaho K.; Hyttinen J.; Miettinen S.; Kellomäki M. Evaluation of Scaffold Microstructure and Comparison of Cell Seeding Methods Using Micro-Computed Tomography-Based Tools. J. R Soc., Interface 2020, 17 (165), 20200102 10.1098/rsif.2020.0102. 32228403 PMC7211473

[ref34] Cengiz I. F.; Oliveira J. M.; Reis R. L. Micro-Computed Tomography Characterization of Tissue Engineering Scaffolds: Effects of Pixel Size and Rotation Step. J. Mater. Sci.: Mater. Med. 2017, 28 (8), 129 10.1007/s10856-017-5942-3. 28721665

[ref35] Krueger E.; Chang A. N.; Brown D.; Eixenberger J.; Brown R.; Rastegar S.; Yocham K. M.; Cantley K. D.; Estrada D. Graphene Foam as a Three-Dimensional Platform for Myotube Growth. ACS Biomater Sci. Eng. 2016, 2 (8), 1234–1241. 10.1021/acsbiomaterials.6b00139. 28164151 PMC5287265

[ref36] Danz R.; Gretscher P. C-DIC: A New Microscopy Method for Rational Study of Phase Structures in Incident Light Arrangement. Thin Solid Films 2004, 462–463, 257–262. 10.1016/j.tsf.2004.05.124.

[ref37] D’Abaco G. M.; Mattei C.; Nasr B.; Hudson E. J.; Alshawaf A. J.; Chana G.; Everall I. P.; Nayagam B.; Dottori M.; Skafidas E. Graphene Foam as a Biocompatible Scaffold for Culturing Human Neurons. R Soc. Open Sci. 2018, 5 (3), 171364 10.1098/rsos.171364. 29657752 PMC5882676

[ref38] Fibroblast’ I. T.; Movat H. Z.; Fernando’ N. V. P. The fine structure of connective tissue. Exp. Mol. Pathol. 1962, 1, 509–534. 10.1016/0014-4800(62)90040-0. 13936387

[ref39] Ruoß M.; Häussling V.; Schügner F.; Damink L. H. H. O.; Lee S. M. L.; Ge L.; Ehnert S.; Nussler A. K. A Standardized Collagen-Based Scaffold Improves Human Hepatocyte Shipment and Allows Metabolic Studies over 10 Days. Bioengineering 2018, 5 (4), 86 10.3390/bioengineering5040086. 30332824 PMC6316810

[ref40] Murphy C. M.; Haugh M. G.; O’Brien F. J. The Effect of Mean Pore Size on Cell Attachment, Proliferation and Migration in Collagen-Glycosaminoglycan Scaffolds for Bone Tissue Engineering. Biomaterials 2010, 31 (3), 461–466. 10.1016/j.biomaterials.2009.09.063. 19819008

[ref41] Moulder J. Handbook of X-Ray Photoelectron Spectroscopy: A Reference Book of Standard Spectra for Identification and Interpretation of XPS Data; Chastain J., Ed.; Physical Electronics Division, Perkin-Elmer Corporation, 1992; Vol. 1.

[ref42] Gaspar B.; Mrzilkova J.; Hozman J.; Zach P.; Lahutsina A.; Morozova A.; Guarnieri G.; Riedlova J. Micro-Computed Tomography Soft Tissue Biological Specimens Image Data Visualization. Appl. Sci. 2022, 12 (10), 4918 10.3390/app12104918.

