# Supplementary material for: Correlative Imaging of Three-Dimensional Cell Culture on Opaque Bioscaffolds for Tissue Engineering Applications
Source: ACS Appl Bio Mater. 2023 Sep 1;6(9):3717–25. doi: 10.1021/acsabm.3c00408 (PMC10521016; doi:10.1021/acsabm.3c00408)
Supplement: Supplementary file 1 — mt3c00408_si_001.pdf [file mt3c00408_si_001.pdf]

Correlative Imaging of 3-dimensional Cell Culture on Opaque Bioscaffolds for Tissue Engineering Applications

Supporting Information

AUTHOR NAMES. Mone't Sawyer,[1] Josh Eixenberger,[2,3] Olivia Nielson,[4] Jacob Manzi,[5] Cadré Francis,[7] Raquel Montenegro-Brown,[6,7] Harish Subbaraman,[5] and David Estrada[3, 6-8]*

AUTHOR ADDRESS.

[1]Biomedical Engineering Doctoral Program, Boise State University, Boise, ID 83725, USA

[2]Department of Physics, Boise State University, Boise, ID 83725, USA

[3]Center for Advanced Energy Studies, Boise State University, Boise, ID 83725, USA

[4]Department of Chemical and Biological Engineering, University of Idaho, Moscow, ID 83844, USA

[5]School of Electrical Engineering and Computer Science, Oregon State University, Corvallis, OR 97331, USA

[6]Center for Atomically Thin Multifunctional Coatings, Boise State University, Boise, ID 83725, USA

[7]Micron School for Materials Science and Engineering, Boise State University, Boise, ID 83725, USA

[8]Idaho National Laboratory, Idaho Falls, ID 83401, USA

[*]Corresponding author: daveestrada@boisestate.edu

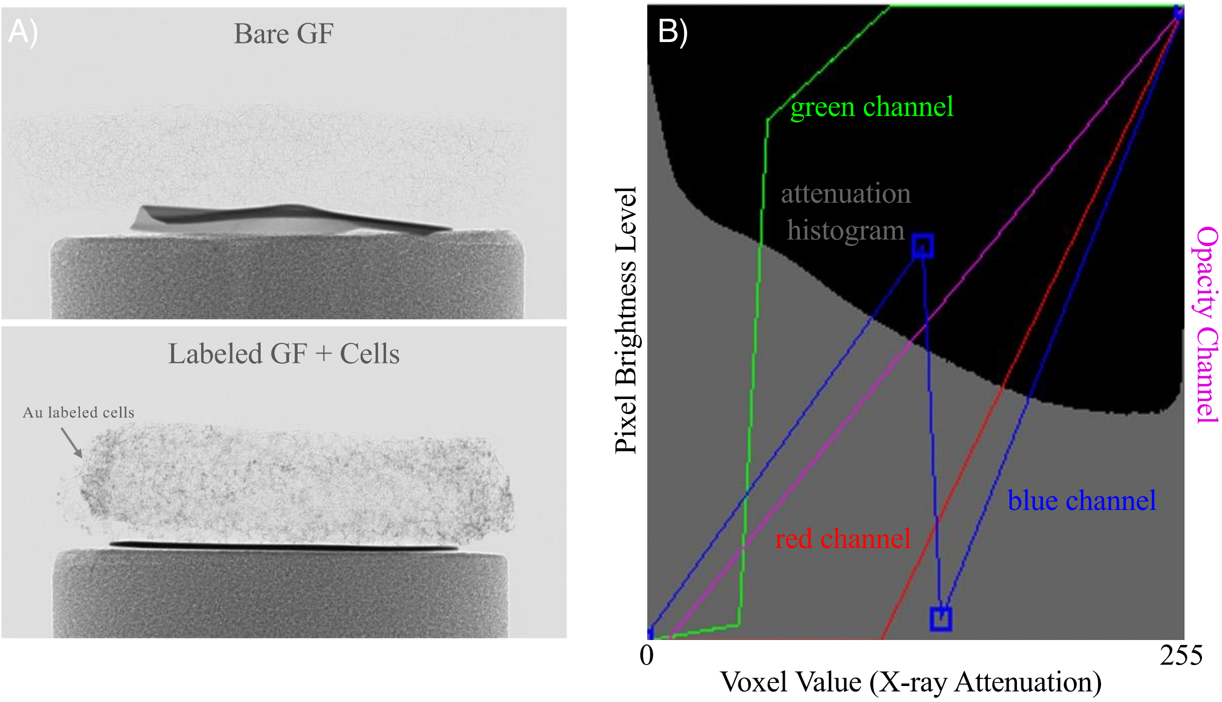

Figure. S1.

Methods for MicroCT imaging and transfer function editing. A) X-ray comparison between bare GF and GF with labeled cells was used to confirm labeling protocol before acquisition where labeled cells are darker in color due to an increase in density from the colloidal gold. B) Transfer function editing with attenuation histogram for CTVox (Opacity, RGB) shadow projections where cells are false colored to gold and GF is colored to grey/black.

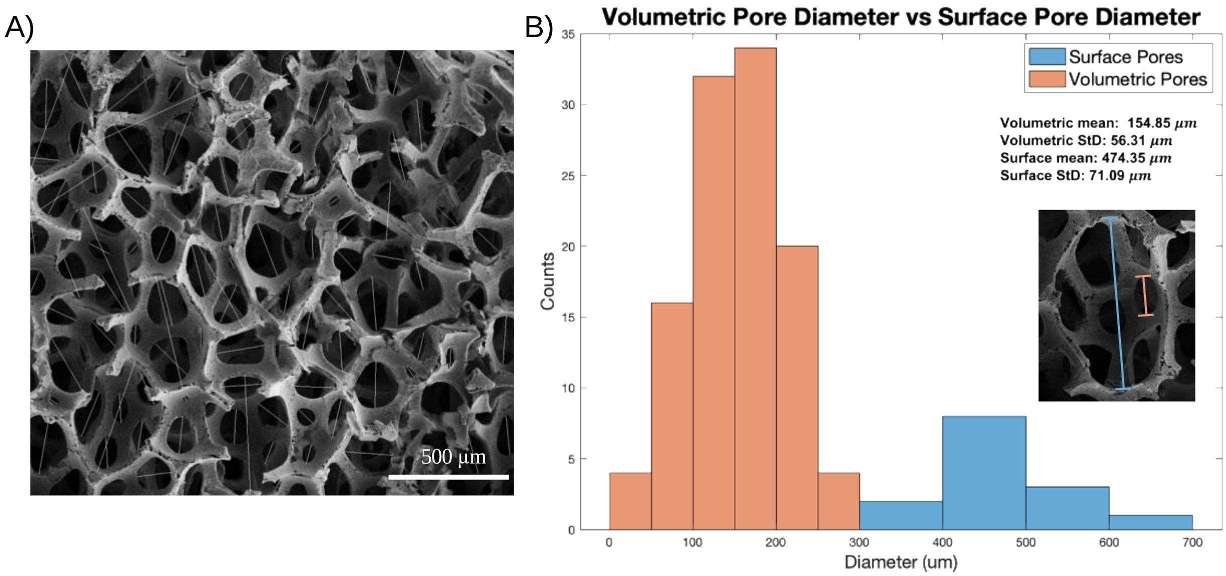

Figure. S2.

Pore size measurements of bare GF. A) Scanning electron micrograph of bare GF indicating the direction that pores were measured utilizing ImageJ. B) Histogram of average pore size for volumetric (orange) and surface pores (blue)

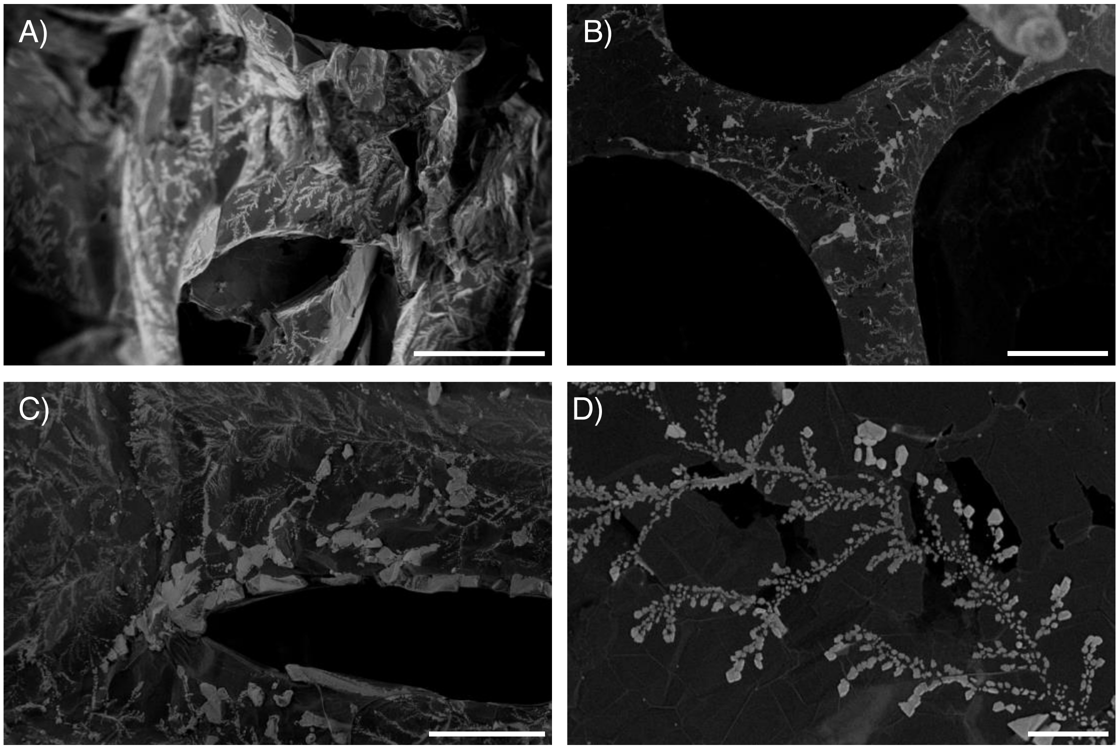

Figure. S3.

When not rinsed with diluted DPBS, salt crystals cover GF branches following cell culture. Scale Bars: A) 50 µm, B) 50 µm, C) 25 µm, and D) 5 µm
